# Ru Catalyst Encapsulated into the Pores of MIL-101 MOF: Direct Visualization by TEM

**DOI:** 10.3390/ma14164531

**Published:** 2021-08-12

**Authors:** Maria Meledina, Geert Watson, Alexander Meledin, Pascal Van Der Voort, Joachim Mayer, Karen Leus

**Affiliations:** 1 Central Facility for Electron Microscopy, RWTH Aachen University, D-52074 Aachen, Germany; mayer@gfe.rwth-aachen.de; 2 Forschungszentrum Jülich GmbH, Ernst Ruska-Centre (ER-C 2), D-52425 Jülich, Germany; 3 Centre for Ordered Materials, Organometallics and Catalysis (COMOC), Department of Chemistry, Ghent University, Krijgslaan 281-S3, 9000 Ghent, Belgium; geert_watson@hotmail.com (G.W.); Pascal.VanDerVoort@ugent.be (P.V.D.V.); Karen.Leus@ugent.be (K.L.)

**Keywords:** TEM, MOF, nanoparticles

## Abstract

Ru catalyst nanoparticles were encapsulated into the pores of a Cr-based metal-organic framework (MOF)—MIL-101. The obtained material, as well as the non-loaded MIL-101, were investigated down to the atomic scale by annular dark-field scanning transmission electron microscopy using low dose conditions and fast image acquisition. The results directly show that the used wet chemistry loading approach is well-fitted for the accurate embedding of the individual catalyst nanoparticles into the cages of the MIL-101. The MIL-101 host material remains crystalline after the loading procedure, and the encapsulated Ru nanoparticles have a metallic nature. Annular dark field scanning transmission electron microscopy, combined with EDX mapping, is a perfect tool to directly characterize both the embedded nanoparticles and the loaded nanoscale MOFs. The resulting nanostructure of the material is promising because the Ru nanoparticles hosted in the MIL-101 pores are prevented from agglomeration—the stability and lifetime of the catalyst could be improved.

## 1. Introduction

Ru-based materials are promising catalysts for various reactions [1,2,3,4]. However, these reactions often run at rather aggressive conditions, for example, at elevated pressures and temperatures. Exposed to high pressure and temperature, the materials tend to lose their nanoparticulate nature and degrade. Approaches to prevent catalyst agglomeration and surface loss are needed to improve their stability and lifetime.

Within this context, metal organic frameworks (MOFs) are perfect candidates to host nanoparticles of a targeted size inside their ordered porous frameworks [5,6,7]. MOFs are highly porous materials built from metal ions or clusters, connected by organic linkers into a three-dimensional structure [8]. The high surface area and tunable porosity make MOFs and MOF-based materials popular in the fields of gas storage [9,10], pollutant adsorption [11,12], and catalysis [13,14,15,16,17]. Besides this, MOFs are also interesting support materials: the nanoparticles become resistant to agglomeration and pose improved stability and longer lifetime when embedded into the porous framework of a MOF [5].

When dealing with such complex systems as nanoparticles@MOFs, it is of paramount importance to be able to characterize the materials at a local scale, for example, to control the positions of the loaded nanoparticles within the porous matrix. Transmission electron microscopy (TEM) provides a unique setup of techniques for visualization and precise investigations of the composite materials [7]. Nevertheless, MOFs are known to be extremely obstinate materials for electron microscopy investigations as they tend to lose their initial structure under the electron beam illumination [18,19]. While operating the TEM for investigations of such fragile materials, the electron dose should be kept minimal to collect trustful and meaningful information. Recently, low dose cameras and detectors are being widely used for the precise imaging of MOFs down to a very local scale [20,21,22].

MIL-101 (Matérial Institut Lavoisier-101) is a Cr-based MOF hosting two types of pores of 29 Å and 34 Å diameter. It is one of the first MOF materials that was directly imaged by TEM, and up until now, it remains a very popular MOF for TEM investigations [23]. This MOF was studied in great detail by several groups applying different techniques, among which are TEM [24], ADF-STEM [25], and iDPC [22]. Several TEM-exploiting studies of the MIL-101 loaded framework were also reported [17,25,26,27,28].

In this work, we applied ADF-STEM imaging for the direct visualization of the MIL-101 pores, hosting the Ru catalyst nanoparticles. Metallic Ru nanoparticles were loaded into the cages of MIL-101 following the wet-chemistry route. To keep the MIL-101 structure in the initial crystalline state during the TEM examination, the electron dose was lowered, and a fast dwell time was used for image acquisition. ADF-STEM confirmed the loading of the crystalline Ru nanoparticles into both types of MIL-101 cages.

## 2. Materials and Methods

MIL-101 was synthesized by using a hydrothermal synthesis approach in which a Teflon-lined container was filled with 4 mmol terephthalic acid, 4 mmol of Cr(NO_3_)_3_·9H_2_O, and 20 mL demineralized water. The mixture was placed in an autoclave and kept at 210 °C for 8 h. The resulting solid was stirred in DMF for 24 h to remove any organic residues. In a later step, the MOF was placed in 1 mol L^−1^ HCl for 12 h to remove any excess Cr salts. In a final step, the material was washed with water until neutral pH, and the purified MIL-101 material was dried under vacuum at 110 °C overnight prior to use.

To introduce Ru, a solution of 287 mg RuCl_3_ in acetone was added to a suspension of 700 mg MIL-101 in acetone. The mixture was left to stir for 24 h after which the RuCl_3_@MIL-101 was collected through filtration.

To reduce the ruthenium precursor, 9.3 mL of a 0.486 mol L^−1^ NaBH_4_ solution was added dropwise to an aqueous dispersion of 700 mg RuCl_3_@MIL-101 in 23.3 mL of demineralized water, after which it was left to stir for 35 min at room temperature. In the end, the Ru@MIL-101 was collected through filtration and was washed successively with demineralized water, ethanol, and acetone.

Annular dark field scanning transmission electron microscopy (ADF-STEM), as well as energy dispersive X-ray (EDX) spectroscopy experiments, were carried out using two different FEI transmission electron microscopes, both operated at an accelerating voltage of 200 kV. FEI Technai Osiris was used to investigate the MIL-101 material; the inner ADF detection angle was 14 mrad. FEI Titan ChemiSTEM was used to investigate the Ru@MIL-101; the inner collection angle ADF detection angle was 54 mrad. The beam current was kept at ~10 pA, and only a single image could be taken before the degradation of the MOF structure. EDX mapping was carried out using a Super-X EDX system on the FEI Titan ChemiSTEM instrument, the beam current was kept at ~80 pA, and the map was recorded in 5 min.

## 3. Results and Discussion

**Figure 1:**
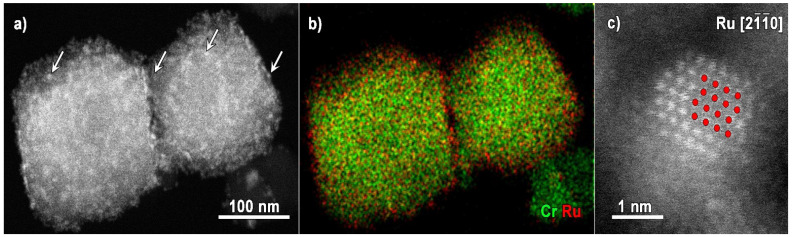
(**a**) Overview ADF STEM image of Ru@MIL-101 particles and (**b**) corresponding EDX map for Cr and Ru, (**c**) ADF-STEM image of a Ru nanoparticle taken along the [$2\bar{1}\bar{1}0$] zone axis with Ru (Ru shown in red) structural model viewed along the [$2\bar{1}\bar{1}0$ ] zone axis.

An overview of the ADF-STEM image of MIL-101 loaded with Ru is presented in Figure 1a. The MIL-101 particles are about 100–200 nm’s in size. Spectroscopy is of great use when investigating such complex systems as MOFs loaded with nanoparticles, as it provides an insight into the spatial distribution of the loading particles elements together with the spatial distribution of the MOF-forming metal. However, due to the extreme beam sensitivity of MOFs, the beam current typically used for EDX acquisition needs to be lowered to avoid fast material degradation and record accurate data. EDX detectors with optimized geometry (when four detectors are positioned around the sample) are greatly helpful as the recorded signal is significantly improved. Figure 1b contains EDX elemental maps for recorded Cr and Ru, keeping a beam current of approximately 20 pA. The bright contrast features in the corresponding ADF-STEM image (Figure 1a, some examples are marked by the white arrows) can be attributed to Ru nanoparticles of a size bigger than the MIL-101 pores. The Ru shell, visible in the EDX map, is related to the nanoparticles, which do not sit inside the pores of the framework but tend to cover it.

An atomic-resolution ADF-STEM image of a Ru nanoparticle taken along the [$2\bar{1}\bar{1}0$] zone axis is placed in Figure 1c. The Ru structural model (235818 card [29], ICSD database), also viewed along the [$2\bar{1}\bar{1}0$] zone axis is overlaid for clarity onto the experimental image, evidencing the metallic Ru crystal structure (space group no. 194, P63/m m c). HR STEM imaging provides a unique insight into the nature of the Ru nanoparticles: due to their small size, no information on the phase could be reached by other techniques, typically used to investigate the crystal structure, like, for example, XRD.

**Figure 2:**
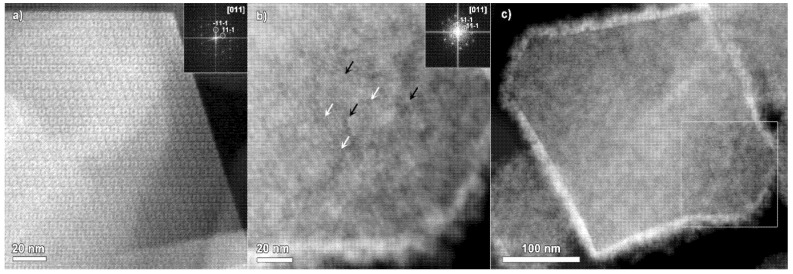
(**a**) ADF-STEM image of MIL-101 crystal taken along the [011] zone axis (Fourier transform pattern is placed in the inset) and (**b**) ADF-STEM image of the Ru@MIL crystal taken along the [011] zone axis of MIL-101 together with the corresponding Fourier transform pattern. The arrows are pointing to some examples of Ru nanoparticles in the smaller (white arrows) and bigger (black arrows) cages of MIL-101. (**c**) ADF-STEM overview of MIL-101 crystal heavily loaded with Ru nanoparticles.

Figure 2a displays an ADF-STEM image of the MIL-101 particle recorded along the [011] zone axis, together with the corresponding Fourier transform pattern in the inset. The ordered porous structure of the material is clearly highlighted. Figure 2b shows an ADF-STEM image of MIL-101 loaded with Ru. The image is also taken along the [011] zone axis of the MIL-101, as evidenced by the Fourier transform pattern in the inset. Both of the ADF-STEM images of MIL-101 and Ru@MIL-101 (Figure 2a,b) were recorded using similar conditions, a beam current of approximately 20 pA, a dwell time of 2 μs, and an operating probe-corrected instrument at 200 kV. The bright contrast layer surrounding the imaged Ru@MIL-101 particle in Figure 2b can be attributed to the layer of Ru nanoparticles, bigger in size than MIL-101 pores. Most probably, on the surface of the supporting MIL-101 particle, a nanoparticulate Ru layer is created. The inhomogeneous contrast within the bulk part of the MIL-101 particle (Figure 2b) could rise from the Ru particles present on the surface of the MIL-101.

The MIL-101 framework hosts two types of pores: smaller ones with a diameter of 29 Å and bigger ones with a diameter of 34 Å. The ADF-STEM mode produces the images with a relatively straightforward way to interpret the contrast. While imaging the empty MIL-101 crystals along the [011] zone axis in ADF-STEM mode (Figure 2a) [25], the smaller pores appear as bright contrast “donuts”. Cr, being the heaviest element in the MIL-101 structure, generates a signal typically higher compared to the lighter elements in the structure (H, O, and C). However, in the image shown in Figure 2b, the situation is opposite to the empty MIL-101. More specifically, ordered darker round contrast features are clearly observed. Compared to the empty MIL-101 crystals, the MIL-101 loaded with Ru demonstrates the inverse contrast while being imaged in ADF-STEM mode. As the atomic number of Cr (Z = 24) is lower than the atomic number of Ru (Z = 44), most of the contrast could be typically attributed to the higher Z Ru loading of the MIL-101 framework. Thus, the smaller pores, in this case, show up as darker contrast “donuts”.

It is of great importance to note that the ADF-STEM imaging clearly confirms that MIL-101 particles remain crystalline after the loading procedure. Both the smaller and larger MIL-101 cages tend to be filled by the Ru catalyst nanoparticles. The white arrows (Figure 2b) point to some examples of bright contrast features with a position in nice agreement with the smaller cages. The black arrows (Figure 2b) indicate some examples of contrast features in the positions of the larger MIL-101 pores.

Figure 2c contains an ADF-STEM overview showing the entire MIL-101 particle, imaged with a lower magnification, in comparison to Figure 2b. The white square indicates the area of the particle, shown in Figure 2b. The image in Figure 2c was taken as the second shot of the particle. The bright contrast layer, confirmed by EDX in Figure 1b to be Ru-based, is most likely covering the MIL-101 crystal. Indeed, the covering layer has a nanoparticulate nature. It is clearly visible, after taking a single image, that the MIL-101 crystal shrunk. This observation is in good agreement with the results described by Yi Zhou and co-authors [22]. The MIL-101 crystal tends to degrade in an inhomogeneous manner across the particle upon electron beam illumination through local structural evolvement. Nevertheless, the ADF-STEM image clearly evidences the typical truncated octahedral shape of the MIL-101 crystal with preferential {111} facets exposed.

**Figure 3:**
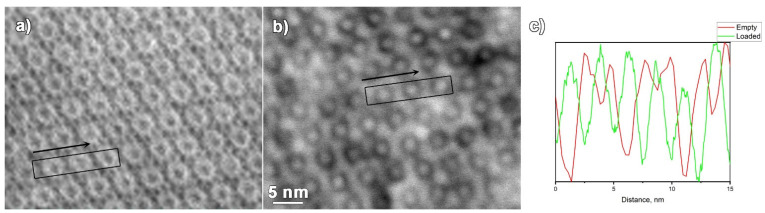
ADF-STEM images of the (**a**) MIL-101 particle and (**b**) Ru@MIL-101 particle, both taken along the [011] zone axis of MIL-101, black boxes and arrows mark the location and direction of the intensity line profiles. (**c**) Intensity line profiles shown in green for the Ru@MIL-101 and red for the MIL-101.

HR ADF-STEM images of the empty MIL-101 and Ru@MIL-101, both taken along the [011] zone axis of MIL-101, are shown in Figure 3a,b. It is clear that the smaller MIL-101 pores, nicely visible in [011] orientation in the case of Ru@MIL-101, show up as dark contrast circles, while the pores in MIL-101 show up as bright contrast circles. The black boxes and the arrows indicate the area and the direction of the line scan profiles placed in Figure 3c. Going along the line profiles of both ADF-STEM images, a clear periodicity can be observed. However, the contrast in the case of the Ru loaded MIL-101 material is the opposite compared to the empty MIL-101, as evidenced by the line profiles. While for the Ru@MIL-101, the peaks in the line profile (Figure 3c, green line) correspond to the Ru-rich areas inside the pores, the peaks in the line profile of the empty MIL-101 (Figure 3c, red line) arise mainly from the Cr-rich areas. In the line profile, the high intensities inside the pores for the Ru@MIL-101 are caused by the Ru nanoparticles sitting in the cages, while for the empty MIL-101, the highest intensities could be attributed to the Cr-based walls of the pores. Together with this, some darker contrast is observed between the smaller pores, which could be attributed in some cases either to the inhomogeneously filled bigger pores or to the contrast arising from Cr-rich supertetrahedra.

## 4. Conclusions

MIL-101 and Ru@MIL-101 materials were investigated on the atomistic scale by ADF-STEM imaging and EDX mapping using low electron dose conditions for accurate and trustful data acquisition with fast dwell time. After the loading procedure, the MIL-101 retains its original crystal structure. The resulting material hosts metallic Ru nanoparticles inside the highly ordered pores, and some Ru nanoparticles are present on the surface of the MIL-101 crystals. Loaded with high Z-number Ru (44 in the periodic table), MIL-101 crystals show the inverse contrast compared to the empty MIL-101 material when imaged in ADF-STEM; most of the contrast arises from Cr (number 24 in the periodic table). The resulting material nanostructure is promising, as the Ru nanoparticles, hosted inside the ordered pores of MIL-101, are prevented from agglomeration. The catalyst material with the obtained structure could pose prolonged stability and lifetime. ADF-STEM imaging and EDX mapping gave a unique and valuable input into the understanding of nanoscale material structure.
